# Chemical Recycling of PLA and Its Copolyesters with Poly(Ethylene Azelate) via Microwave-Assisted Alkaline Hydrolysis and Enzymatic Hydrolysis

**DOI:** 10.3390/polym17101374

**Published:** 2025-05-16

**Authors:** Rafail O. Ioannidis, Nikolaos D. Bikiaris, Evangelia Vouvoudi, Alexandra Zamboulis, Nikolaos Nikolaidis, Dimitrios N. Bikiaris

**Affiliations:** Laboratory of Polymer and Colors Chemistry and Technology, Aristotle University of Thessaloniki, GR-541 24 Thessaloniki, Greece; rafailio@chem.auth.gr (R.O.I.); nikompik@pharm.auth.gr (N.D.B.); evouvoud@chem.auth.gr (E.V.); azamboulis@gmail.com (A.Z.); nfnikola@chem.auth.gr (N.N.)

**Keywords:** poly(lactic acid), poly(ethylene azelate), copolyesters, microwave irradiation, recycling, alkaline hydrolysis, enzymatic hydrolysis

## Abstract

Poly(lactic acid) (PLA) is a widely used biobased polyester which can be derived from renewable resources. Due to its excellent properties, it has already been adopted in various industrial sectors. While PLA is compostable, its degradation to the environment is very slow, necessitating the development of efficient recycling methods. This study focuses on the chemical recycling via microwave-assisted alkaline hydrolysis of PLA and its copolymers with poly(ethylene azelate) (PEAz), aiming to recover both carboxylic acid monomers: lactic acid and azelaic acid. Moreover, our method tunes the degradation of PLA via the synthesis of the novel aliphatic PLA-based copolyesters, targeting engineering-like applications, specifically in the field of printed electronics. Various process parameters were analyzed, including the temperature and the duration of the experiments as well as different phase transfer catalysts. Complete degradation was achieved at low temperatures (110–125 °C) and short times (12–15 min) for the PLA-based copolyesters, offering significant environmental benefits, as considerably less energy is consumed compared to chemical conventional methods. So, by changing the composition of the copolyesters through the incorporation of PEAz blocky segments, the ester bonds became more susceptible to hydrolysis under alkaline conditions assisted with microwave irradiation. Additionally, enzymatic hydrolysis was also studied in parallel for comparative purposes, revealing low degradation rates, thus establishing the microwave-assisted alkaline hydrolysis as a solid and reliable method for tuning the degradation of PLA-based materials.

## 1. Introduction

The emergence of screen-printed electrodes (SPEs) and, generally, the field of printed electronics has offered unlimited possibilities for their application in the analysis of biological fluids such as blood, plasma, tears, or sweat. SPEs are devices that can be produced using different inks on a plethora of substrates, i.e., ceramic or plastic, utilizing printing techniques. Their inexpensive nature has enabled a high-volume production of single-use sensors with high repeatability and reliability [1,2,3,4]. Common plastic substrates include poly(vinyl chloride) (PVC) and poly(ethylene terephthalate) (PET), poly(ethene naphthalate) (PEN), and polyimide (PI), all of which are derived from petroleum-based resources. However, their non-biodegradable and synthetic character contributes to the accumulation of plastic waste, posing a threat to the environment [5]. The printed electronics market value was estimated at USD 55 billion in 2020 and is anticipated to reach USD 300 billion by 2030. Consequently, electronic biosensors that reach their end-of-life (EoL) may contribute to a significant amount of electronic waste (“*e*-waste”) [6].

To address these issues, a shift toward biobased, biodegradable, and non-toxic polymers is urgent [7,8,9,10,11], especially considering the increasing demand for this type of materials. Palmieri et al. [12] prepared biodegradable, water-resistant, and flexible substrates based on ethyl cellulose for electrochemical printed platforms. Pei-Leun Kang et al. [13] fabricated screen-printing substrates from biodegradable, native, and modified gelatin films. The group of Mettakoonpitak [14] investigated polycaprolactone (PCL) for simple screen-printing to fabricate microfluidic, paper-based analytical devices (mPADs). Poly(lactic acid) (PLA) is a promising biodegradable and biobased polymer for use as an environmentally friendly alternative in SPEs. Its monomers (e.g., lactide or lactic acid) are derived from bioresources via fermentation. PLA can be synthesized via ring-opening polymerization (ROP) or polycondensation reactions [15,16].

Another issue currently related to plastic usage is the pollution that is caused by end-of-life mismanagement [17,18]. Thus, apart from the transition toward biobased materials, there is also a necessity to consider the EoL handling of these materials and ensure there are existing routes of effective disposal to make plastic usage sustainable and reduce its environmental footprint. The future of plastic utilization should be grounded in a circular economy model, wherein the material’s value is preserved beyond its initial use [19]. Fossil-derived substrates, such as PET, present significant environmental concerns due to their persistence in the environment, dependence on non-renewable resources, and resistance to degradation under mild chemical conditions, which hinder efficient recycling. With respect to functional performance these materials are typically unsuitable for low-energy recycling methods and contribute substantially to the accumulation of electronic waste [20,21,22].

The traditional and most employed method of plastic management is mechanical recycling. Still, one of its main disadvantages is the property deterioration of the recycled products, especially for aliphatic polyesters. Hence, more effective approaches are essential. Lately, chemical recycling of high molecular weight polymer wastes into smaller-sized constituents has been investigated, as these constituents have a great potential as starting materials for other value-added products [23,24,25,26,27]. Hirao and his coworkers [28] were the first to test alcoholysis of polyester under microwave irradiation, in which its products were compared with those from conventional heating methods. Moreover, Nim et al. [29] developed a microwave-assisted alcoholysis process using diols and a titanium (IV) butoxide (TBT) catalyst for the recycling of PLA. The effects of the types of diols and PLA/diol feeding ratios on the properties and structures of the obtained alcoholized products were investigated by using propane-1,3-diol (PDO), butane-1,4-diol (BDO), and ethylene glycol (EG). Siddiqui et al. [30] also depolymerized PLA in a microwave reactor through phase transfer catalyzed alkaline hydrolysis. They achieved more than 90%, using hexadecyltrimethylammonium bromide as a phase transfer catalyst in a 10% *w*/*v* NaOH medium at 100 °C for 10 min irradiation time.

Microwave irradiation can be considered one of the most suitable and crucial tools for accelerating the degradation of PLA, as conventional chemical hydrolysis methods (i.e., enzymatic or chemical hydrolysis under alkaline or acidic conditions without the presence of microwave irradiation) often require several hours to days at high temperatures and typically yield only moderate results. By implementing microwave irradiation, the reaction time and energy consumption can be rapidly decreased, as microwave heating is quasi-instantaneous, uniform, and fast. So, the aforementioned method can be considered environmentally preferable compared to the traditional recycling ones (i.e., mechanical recycling). Moreover, chemical recycling facilitated by microwave-assisted heating presents an opportunity to achieve a potentially closed-loop recycling process [31,32,33,34].

In this study, novel PLA copolyesters based on PLA and poly(ethylene azelate) (PEAz) were used to modulate PLA degradation, alongside a straightforward approach for recycling substrates intended for printed electronics applications. Furthermore, the objective of developing PLA-based copolyesters was to overcome the limitations in the overall mechanical performance of PLA—specifically its low elongation—while preserving its favorable thermal properties. Thus, bio-based, tough, and flexible PLA/PEAz blocky copolyesters were synthesized via the ROP of L-lactide, using PEAz as a macroinitiator [35].

Herein, recycling studies were conducted on novel PLA blocky copolyesters based on PEAz [35], aiming at the recovery of the lactic acid and azelaic acid monomers. PLA/poly(ethylene azelate) (PEAz) blocky copolyesters, produced from 100% biobased monomers, were developed as substrates for printed electronics to overcome the poor heat resistance and inherent brittleness of PLA [36,37]. Herein, a process for the chemical recycling of these copolymers was developed based on a microwave-assisted alkaline reaction. Hexadecyltrimethylammonium bromide (HTMAB) proved to be an efficient phase transfer catalyst. Microwave irradiation was applied as a heating source for decreasing the reaction time and enhancing the reaction efficiency. Lactic acid and azelaic acid were successfully recovered. To compare the degradation rates of PLA and PLA-based copolyesters, enzymatic hydrolysis was also performed, highlighting the importance of microwave-assisted alkaline hydrolysis.

## 2. Materials and Methods

### 2.1. Materials

Azelaic acid (AzA) (purity > 99.0%) was supplied from Fluka (Steinheim, Germany); 1,2-ethanediol (purity, >99.8%), 1-dodecanol, and tin(II) 2-ethylhexanoate (Sn(Oct)_2_ were purchased from Aldrich Co., (London, UK). Titanium (IV) butoxide (Ti(OBu)_4_ (TBT), purity: >97.0%) was supplied by Sigma Aldrich Chemical Co. (Steinheim, Germany), and the LA (99.9%) was purchased from PURAC Biochem BV (Gorkum, The Netherlands). The phase transfer catalysts used were as follows: (2-dimethylaminoethyl) triphenylphosphonium bromide [(2DME)-TPPB] (Lancaster, UK), hexadecyltrimethylammonium bromide (HTMAB) obtained from Fluka Chemicals, and aqueous solution of hexadecyltrimethylammonium chloride (HTMAC), 25 wt% from Aldrich. All other materials and solvents used were of analytical grade.

### 2.2. Synthesis of PLA, PEAz, and Their Copolyesters

The PLA and PLA-based copolyesters were synthesized via ring-opening polymerization of L-lactide, where PEAz was synthesized [38] via a two-stage melt polycondensation procedure. A detailed procedure was described in our recently published work [35]. In brief, PLAs were synthesized by *in situ* ring-opening polymerization (ROP) of L-lactide in the presence of Sn(Oct)_2_ (400 ppm). Dodecanol (0.05 g/mL in acetone) was added as a co-initiator. The polymerization was carried out at 160 °C for 2 h, at 350 rpm under continuous N_2_. After 2 h, the temperature was increased to 180 °C at 400 rpm for additional 15 min. The polymerization was terminated by rapid cooling of the flask to room temperature. The PLA-based copolyesters were synthesized similarly but in the presence of poly(ethylene azelate) (PEAz), instead of dodecanol, which acted as macroinitiator. In total, four different copolyesters were synthesized, namely: 97.5-2.5, 95-5, 90-10, and 80-20.

### 2.3. Alkaline Hydrolysis Assisted with Microwave Irradiation

**Figure 1:**
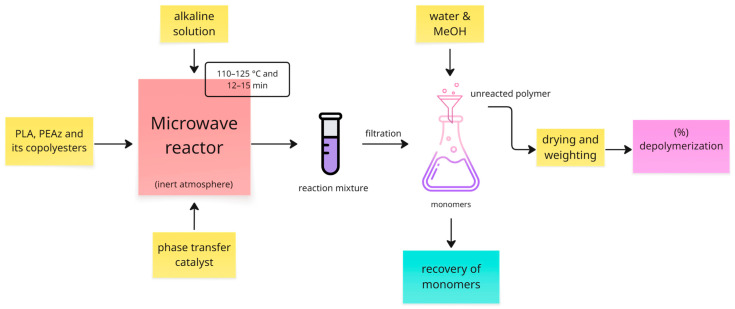
General experimental procedure of microwave-assisted alkaline hydrolysis of PLA and its copolyesters.

The hydrolytic depolymerizations of PLA samples were conducted in a microwave reactor (model Discover, CEM Corporation, Tokyo, Japan), equipped with a digital temperature control system and pressure sensors. The samples were placed into a 10 mL glass tube. NaOH solution was prepared (10% *w*/*v*) as the medium for the decomposition reactions. The PLA samples 0.5 g together with 4 mL of NaOH solution were added to the reactor together with a magnetic stirrer. The tubes were filled with nitrogen to retain an inert atmosphere, sealed, and then heated up for different time intervals. In some experiments, 0.05 g of HTMAB (1:10 *w*/*w* PLA) or other phase transfer catalyst (PTC) were added to the reactor in order to compare different catalysts. When the set temperature was achieved, the reaction time began, and the depolymerization of PLA followed. After the reactions, the tube was automatically cooled, and the reaction mixture was filtered to remove the unreacted PLA residues. The residues were washed with distilled water, followed by methanol to remove excess of NaOH. The final unreacted PLA was dried in a vacuum oven at 60 °C and weighed. The same procedure was followed for the PLA-based copolyesters and PEAz. Figure 1 presents a general procedure of alkaline hydrolysis. The degradation rates of the samples were calculated based on the following equation:

(1) $$\%\;Depolymerization=\frac{W_{initial}-W_{final}}{W_{initial}}$$

where W*_initial_* and W*_final_* refer to the initial and final weights of the samples, respectively.

During the depolymerization reactions of the PLA-based materials, the average detected microwave power ranged between 2 and 4 Watts. Temperature regulation was achieved using an internal control system.

### 2.4. Recovery of Lactic Acid and Azelaic Acid

**Figure 2:**
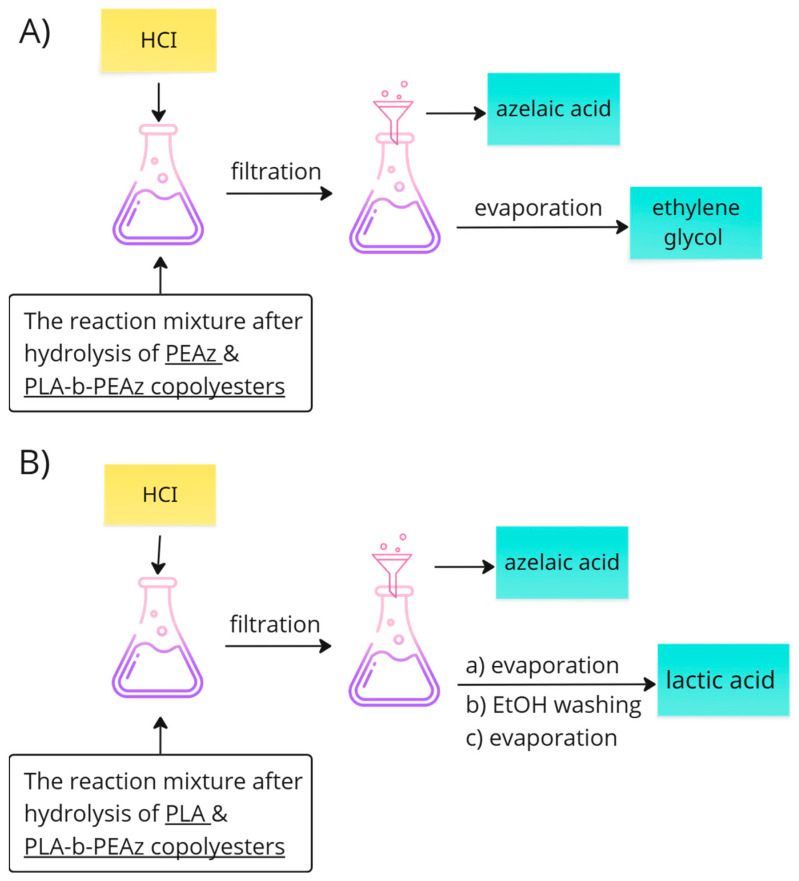
General scheme for the recovery of monomers.

After the depolymerization reactions of the (co)polymers (for the fully degraded samples), each mixture (which contained the oligomers, monomers, the catalyst, and excess of the NaOH solution) was collected for further experiments, and specifically for the recovery of lactic acid and azelaic acid. Figure 2 presents a general flow of recovering the monomers.

- In the case of poly(ethylene azelate), the HCI solution was added in order to reach the pH value close to 1–2. In acidic conditions the precipitation of azelaic acid immediately occurred and the acid was collected through filtration. After the evaporation of the solvents, ethylene glycol was retrieved, but only in the case of ethylene glycol.
- In the case of PLA and its copolyesters, to remove the (co)-polymer residues, the filtrate was acidified, causing azelaic acid to precipitate. It was then collected by filtration, and the filtrate was concentrated. Lactic acid was subsequently collected after the addition of EtOH (by concentration).

### 2.5. Enzymatic Hydrolysis

For the enzymatic degradation studies, thin films produced via compression molding were cut into squares approximately 1.5 cm × 1.5 cm in size and placed in test tubes containing 10 mL of phosphate buffer solution (pH 7.4) supplemented with 0.01 mg/mL lipase from Pseudomonas cepacia and 0.1 mg/mL lipase from Rhizopus oryzae. The tubes were then incubated at 37.0 ± 1 °C in an oven for one month while the media were replaced every 3 days. Every 5 days, the samples were removed from the flasks, washed with distilled water, dried under vacuum, and weighed until constant weight. The degree of enzymatic hydrolysis was estimated from the mass loss of the samples. Blank incubations were also carried out with the samples in the same buffer without enzyme addition. The degree of biodegradation was estimated by the sample weight loss, according to the following equation:

(2) $$Mass\;loss\;\left(\%\right)=\frac{W_{o}-W_{i}}{W_{o}}$$

Each sample was hydrolyzed in triplicate, and the mean value was calculated.

The samples for enzymatic hydrolysis were prepared by compression molding using an Otto Weber, Type PW 30 hydraulic press connected with an Omron E5AX Temperature Controller (Kyoto, Japan), at a temperature of 155–165 °C (depending on the sample) and a pressure of 100 mbar. After melt-pressing, the films cooled rapidly at room temperature. All the films had a similar thickness of approximately 0.30 ± 0.05 mm.

### 2.6. Characterizations

#### 2.6.1. GPC—Gel Permeation Chromatography

GPC was used in order to measure the molecular weight of the –(co)-polymers, with a Waters 600 high-performance liquid chromatographic pump, with different columns (Waters Ultrastyragel, Milford, CT, USA) HR-1, HR-2, HR-4E, HR-4, and HR-5, and a refractive index detector (Shimadzu RID10A, Kyoto, Japan). A detailed procedure was described in our recently published work [28]. The measurements were performed in triplicate.

#### 2.6.2. NMR—Nuclear Magnetic Resonance

The NMR spectra were conducted in deuterated dimethylsulfoxide, DMSO for the structural study of lactic acid, azelaic acid, ethylene glycol and the phase transfer catalyst. An Agilent 500 spectrometer was used (Agilent Technologies, Santa Clara, CA, USA) at room temperature. Spectra were calibrated using the peaks of the residual solvents. The measurements were performed in triplicate.

#### 2.6.3. DSC—Differential Scanning Calorimetry

For the DSC measurements, a PerkinElmer Pyris Diamond DSC differential scanning calorimeter was used and calibrated with three metals, pure indium, zinc, and tin standards, in an inert atmosphere (N_2_). All the samples were measured around 5 mg ± 0.1 and sealed in aluminum pans.

Regarding the heating scans, a simple heating step was conducted (20 °C/min) in order to measure the melting temperature of the materials (T_m_). The measurements were performed in triplicate.

#### 2.6.4. SEM—Scanning Electron Microscopy

SEM analysis was performed with a Tescan Vega Compact (Tescan group a.s., Brno, Czech Republic) instrument at an accelerating voltage of 5–10 keV.

## 3. Results and Discussion

### 3.1. Synthesis and Microwave Hydrolysis of PLA and Its PEAz Copolymers

**Figure 3:**
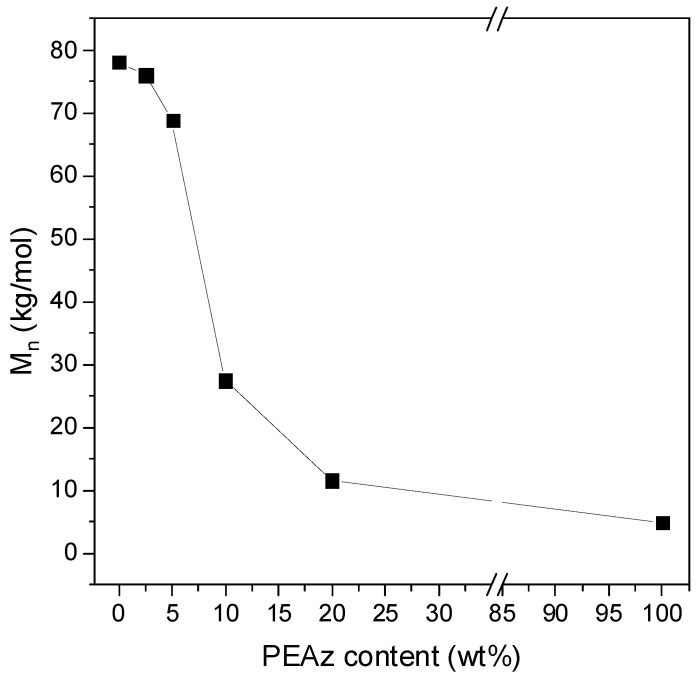
$\bar{M_{n}}$ of PLA, PEAz, and its copolyesters. The data used in the generation of Figure 3, reproduced from Ref. [35] with permission from the Royal Society of Chemistry.

PLA and PLA block copolymers based on PEAz were synthesized for the first time by our group, targeting engineering applications (i.e., printed electronics, biosensors), as it was mentioned in our recently published work [35]. GPC measurements confirmed the high molecular weight of the synthesized materials (Figure 3). From the calculated results, it was clear that while the amount of PEAz increased, the molecular weight (specifically, the number average molecular weight—$\bar{M_{n}}$) of the copolyesters decreased. This was expected, since the -OH groups in PEAz can act as initiators for the lactide ring opening polymerization. As demonstrated in our previous NMR analysis, all copolyesters have a blocky structure [35].

**Figure 4:**
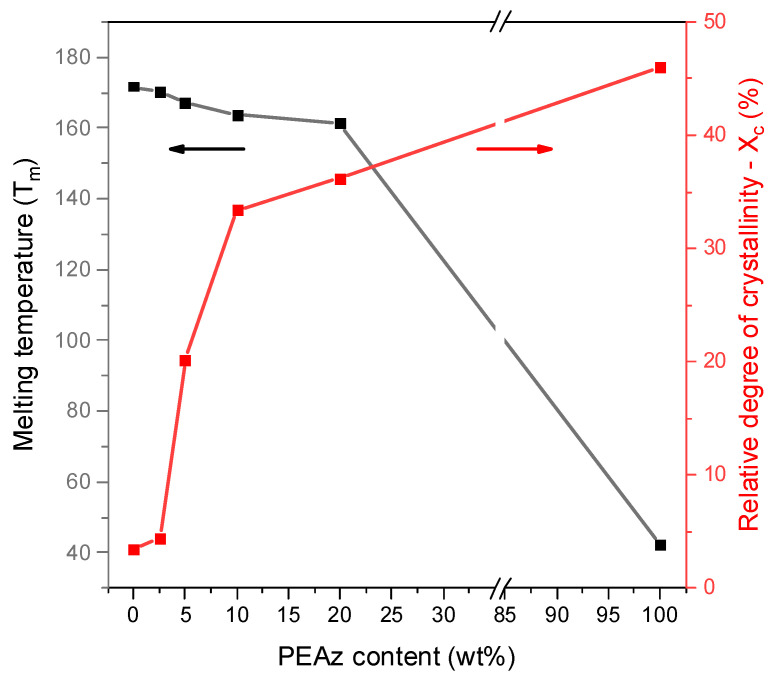
Melting temperature and relative degree of crystallinity of PLA and its copolymers, during the first DSC scan, shown as a function of PEAz content. The data used in the generation of Figure 4 are reproduced from Ref. [35] with permission from the Royal Society of Chemistry.

The investigation of the molecular weight and thermal properties (Figure 4) (especially the relative degree of crystallinity) of the materials was critical because the degradation of PLA and PLA-based copolymers is mostly affected by the interactions among the macromolecular chains and the amorphous regions. In the case of PLA/PEAz blocky copolyesters, the incorporation of PEAz blocky segments into the PLA macromolecular architecture resulted in a decrease in the melting temperature, which is attributed to the formation of less ordered PLA crystalline domains relative to those observed in neat PLA. Moreover, the relative degree of crystallinity increased because of the additional crystal regions associated with the formation of PEAz crystalline domains [35,38,39,40].

Concerning chemical hydrolysis reactions, a suitable phase transfer catalytic system should be used. Preliminary experiments were conducted using different catalytic systems and mediums for the alkaline hydrolysis experiments assisted with microwave irradiation (Table 1). Thus, three different catalysts were used (1/10 *w*/*w* PLA), using NaOH 10% *w*/*v* as a medium, keeping constant the temperature and the time of the reactions. Once the maximum depolymerization was achieved for a specific catalyst, an additional experiment was conducted using as a medium only an aqueous solution to highlight the impact of the alkaline condition.

The effect of the catalyst is presented in the next table, showing the degradation of the sample at 80 °C for 5 min. The most suitable catalyst for our system was hexadecyltrimethylammonium bromide (HTMAB), achieving almost 70% depolymerization, compared to the other catalysts tested. The presence of the phase transfer catalyst was crucial for effectively transferring hydroxide ions from the aqueous to the organic phase. Thus, in this way, it was possible for the ions to attack the PLA’s ester bonds more efficiently, improving the reaction rate. Moreover, the alkaline conditions were essential for the best possible degradation reaction of PLA, since the use of aqueous solution alone (Table 1) did not sufficiently facilitate the depolymerization of PLA. As a result, the presence of hydroxide ions in the alkaline solution was indispensable.

Different degradation conditions (NaOH concentration, catalyst amount, time, and temperature) were optimized for PLA (Table 2, Table 3, Table 4 and Table 5). Table 2 and Table 3 show the impact of the concentration of the alkaline solution and the amount of catalyst, respectively. The highest degradation rate of PLA was achieved in the case of NaOH 10% *w*/*v*. Low concentrations of the alkaline solution resulted in insufficient reactions rate, while, on the other hand, at higher concentrations, the solubility of the depolymerization products was lower, and the degradation of the sample was hindered. Moreover, the optimal catalyst amount was 0.05 g. A further increase did not improve significantly the degree of depolymerization (Table 4).

Table 4 and Table 5 show the effect of temperature and time on the degradation rate of PLA samples under microwave irradiation. As expected, the depolymerization of PLA increased with an increasing temperature, reaching almost 100% at 120 °C after 10 min. Regarding the degradation rates as a function of time (Table 5), PLA fully degraded at 125 °C after 15 min. Interestingly, after just 2 or 5 min, the degradation rate was very high (over 60%), meaning that the microwave irradiation had a significant impact on the depolymerization reactions of the samples.

Regarding hydrolysis of PLA, similar results were reported compared to the work of Siddiqui and his co-workers [30]. The importance of heating the system with microwave irradiation by the presence of a transfer phase catalyst was necessary especially by the time the hydrolysis takes place by conventional heating without the presence of any catalyst. In detail, in an aqueous solution, high degradation rates of PLA occurred at higher temperatures (160–180 °C) for 150–180 min [41].

**Figure 5:**
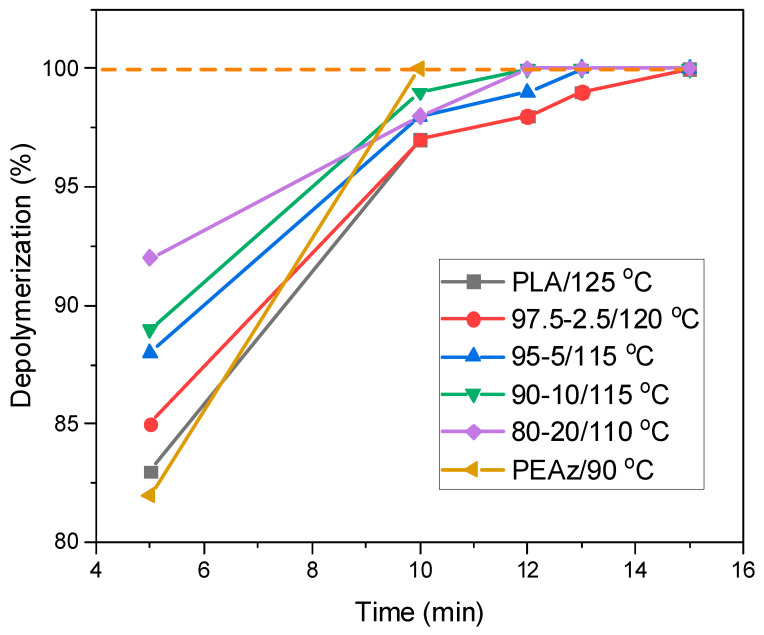
Depolymerization of the samples as a function of time at specific temperatures. The dash line indicates the absolute dissolution (tangent line).

In the case of PLA/PEAz copolyesters, the same procedure was followed in order to find the best conditions for their complete hydrolysis. Figure 5 presents the depolymerization of the samples as a function of time at specific temperatures. All the samples were degraded at relatively mild conditions. As the content of the second comonomer (PEAz) was increased, the time and temperature necessary for complete depolymerization decreased. For instance, the 80-20 PLA/PEAz blocky copolyester fully depolymerized at a temperature 15 °C lower and 5 min faster compared to PLA. In other words, PLA degradation was tuned by the presence of PEAz blocky segments. It is worth mentioning that the PEAz homopolyester fully degraded at 90 °C in just 10 min, and that happened due to its significantly lower molecular weight compared to the other materials (Figure 3).

The PLA/PEAz blocky copolyesters exhibited rapid and efficient degradation via microwave-assisted alkaline hydrolysis, promoting sustainable alternatives to conventional materials, addressing the issue of electronic waste accumulation. By enabling controlled and accelerated depolymerization under relatively mild conditions, the PLA-based copolyesters could contribute to the development of electronic devices with reduced long-term environmental impact [42,43,44,45].

**Figure 6:**
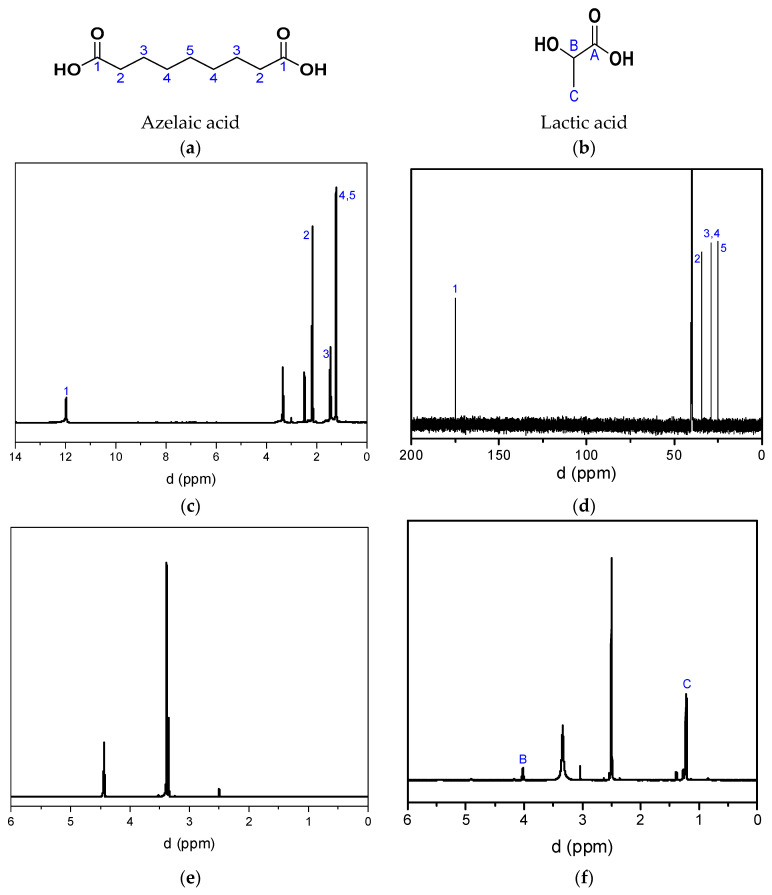

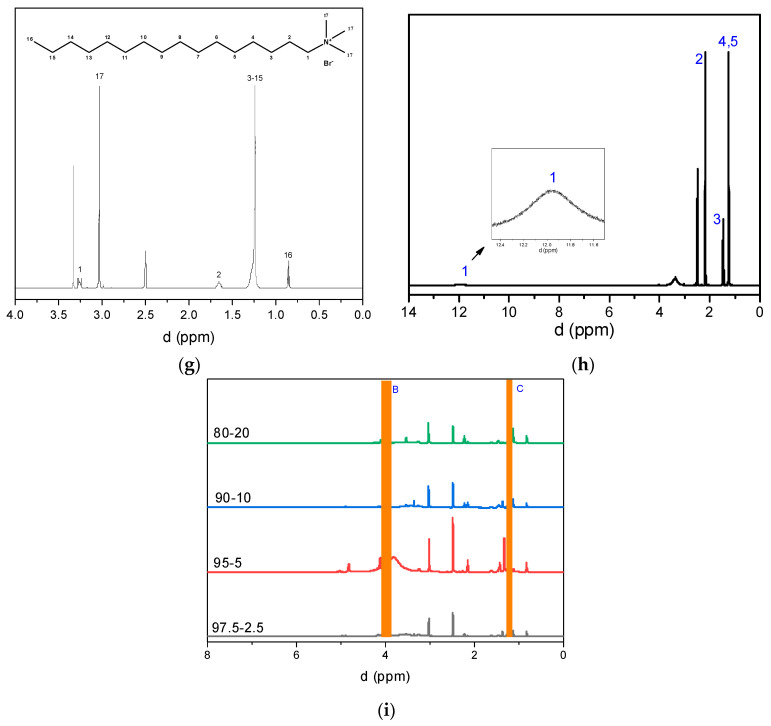
(**a,b**) Chemical structure of azelaic acid and lactic acid. NMR spectra of isolated monomers retrieved after polyester hydrolysis. Spectra are recorded in DMSO-d6. The solvent gives a resonance signal at 2.50 ppm in the ^1^H spectra and 39.52 ppm in the ^13^C spectra. Additionally, residual water appears at 3.33 ppm in the ^1^H spectra. (**c**) ^1^H and (**d**) ^13^C spectra of azelaic acid after complete depolymerization of PEAz. In the ^1^H spectra: 1.24 ppm -CH_2_- 4/5, 1.46 ppm -CH_2_- 3, 2.17 ppm -CH_2_- 2, 11.97 ppm -COOH (1). In the ^13^C spectra, 24.8 ppm -CH_2_- 5, 28.86 ppm -CH_2_- 4, 28.90 ppm -CH_2_- 3, 34.0 ppm -CH_2_- 2, 174.9 ppm -COOH (1). (**e**) ^1^H spectra of ethylene glycol after PEAz depolymerization: 3.39 ppm -CH_2_-, 4.43 ppm -OH. (**f**) ^1^H spectra of lactic acid after PLA depolymerization. 1.27 ppm -CH_3_ C, 3.40 ppm -OH, 4.02 ppm -CH- 3. (**g**) ^1^H NMR spectra of the phase transfer catalyst. (**h**) ^1^H NMR spectra of azelaic acid after depolymerization of PLA/PEAz 80-20 copolyester and (**i**) ^1^H NMR spectra of lactic acid after depolymerization of PLA/PEAz copolyesters.

After the depolymerization of the homo- and copolyesters, azelaic acid and lactic acid recovered, as evidenced by the respective NMR spectra of the isolated monomers in Figure 6. In the case of PEAz homopolymer, the addition of HCl solution to the filtrate collected after the filtration to remove PEAz residues resulted in the precipitation of azelaic acid. Azelaic acid (Figure 6c,d) was isolated by filtration, and ethylene glycol was retrieved (Figure 6e) from the filtrate. Azelaic acid was also isolated in the case of PLA/PEAz 80-20 copolyester (Figure 6h). For the hydrolysis of PLA, after the removal of PLA residues, the filtrate was acidified and concentrated, and lactic acid was extracted (Figure 6f) from the solid residue containing EtOH. Finally, these two protocols were combined for copolyesters. Indeed, after filtration to remove the polymer residues, the filtrate was acidified; azelaic acid precipitated and was filtered off; the filtrate was concentrated, and lactic acid was extracted (Figure 6i) by EtOH washing. Remaining traces of phase transfer catalyst were detected almost in all cases. Figure 6g also presents the ^1^H NMR spectra of the used phase transfer catalyst.

Chemical recycling can be considered one of the most efficient approaches among the recycling techniques. Especially compared to the mechanical recycling, which is the most commonly used method in industrial sectors. However, one common issue is considered the deterioration of the properties of the materials at the end of the mechanical recycling cycle [46,47].

### 3.2. Enzymatic Hydrolysis of PLA and Its PEAz Copolymers

**Figure 7:**
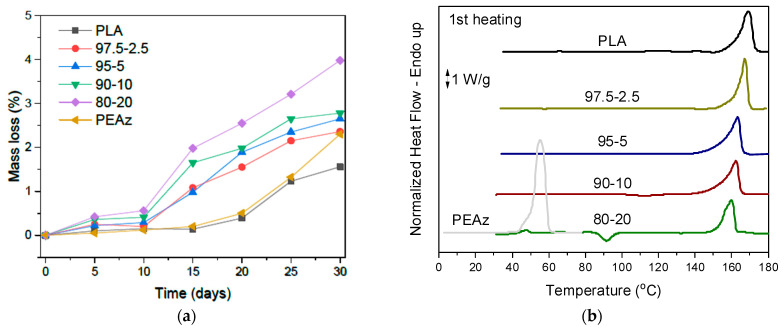
(**a**) Enzymatic hydrolysis of the homopolyesters and copolyesters, and (**b**) the DSC curves after 30 days of enzymatic hydrolysis of the as-received samples, with a heating rate of 20 °C/min.

**Figure 8:**
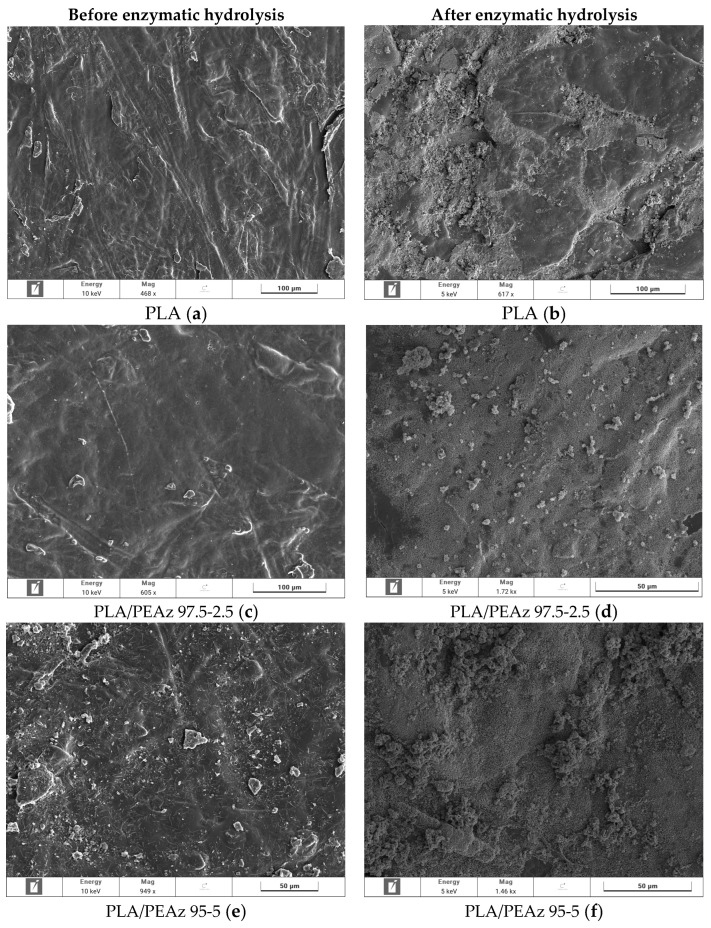

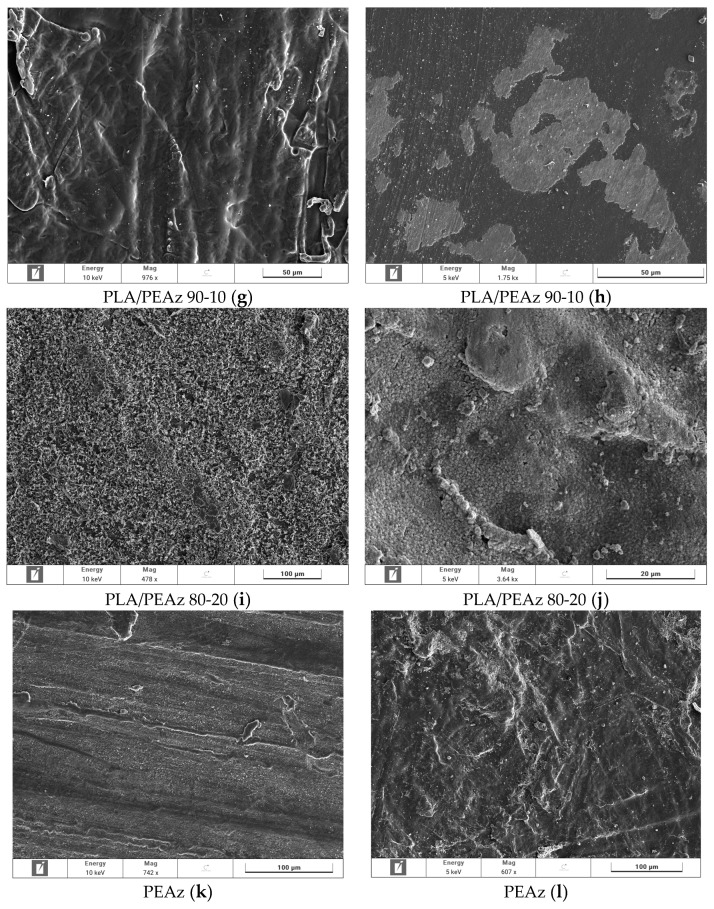
SEM micrographs of the surfaces of the samples before and after 30 days of enzymatic hydrolysis. The magnification varied from 20 and 50 to 100 μm.

Enzymatic hydrolysis was also performed in order to test the degradability of the PLA and PLA-based copolymers in the presence of enzymes, for comparison reasons with the alkaline hydrolysis assisted with microwave irradiation. Enzymatic hydrolysis of the copolymers was conducted in aqueous PBS buffer using lipases from Rhizopus Arrhizus and Pseudomonas Cepacia lipases, two of the most efficient enzymes for polyester degradation. at 37 °C [48,49]. Enzymatic degradation was determined by measuring the mass loss (Equation (2)) of specimens of similar size and weight. The specimens were taken out every 5 days for 1 month, washed with water, and dried under vacuum. All the materials have shown potentially biodegradable behavior, but not enough (Figure 7a). On the other hand, the mass loss of the materials followed the trend of PEAz content, meaning that the copolyesters can tune also the degradation of PLA [48]. DSC scans after 30 days of enzymatic hydrolysis (Figure 7b) revealed that the melting temperatures of the samples did not change compared with the initial DSC measurements (Figure 4) [35]. The low mass loss profile of the samples can be attributed to the high values of the relative degree of crystallinity (Figure 4), where hydrolysis is hindered by the presence of crystalline regions. Furthermore, the high molecular weight of the materials and the presence of the side methyl group in the structure of the PLA blocky segments hindered the enzymatic hydrolysis [16,40,48]. For these reasons, the use of microwave irradiation was considered mandatory for effective hydrolysis [30]. Moreover, the surface of the samples was investigated via SEM (Figure 8), where their morphology did not significantly alter. Only surface erosion was observed, with no evidence of bulk degradation and without any significant morphological changes that would indicate efficient enzymatic hydrolysis. The presence of crystalline regions which hinder the diffusion of water and the enzymes within the PLA matrix, thereby limiting their absorption and making bulk erosion unlikely [49,50]. At this point, the importance of microwave-assisted alkaline hydrolysis should be noted, compared with other degradation approaches. Specifically in this study, the degradation rates of enzymatic hydrolysis were significantly lower in comparison with alkaline hydrolysis assisted with microwave irradiation [51,52,53].

## 4. Conclusions

Microwave-assisted alkaline hydrolysis proved to be one of the most efficient recycling methods for not only accelerating the degradation of PLA and PLA-based copolyesters but also a facile way to recover the monomers of the materials. The depolymerization of the samples occurred at relatively mild conditions, where the second comonomer of the PLA/PEAz copolyesters played a significant role in the degradation of PLA. Specifically, PLA-based materials were depolymerized completely in an efficient and rapid manner at 110–125 °C within 12–15 min (depending on the PEAz composition) in the presence of HTMAB, which acted as a phase-transfer catalyst, significantly accelerating the degradation reactions. The presence of the blocky poly(ethylene azelate) segments within the structure of PLA-based copolyesters facilitated the depolymerization reactions, decreasing slightly both the temperature and time of alkaline hydrolysis. For comparison reasons, enzymatic hydrolysis experiments revealed low degradation rates, highlighting the importance of microwave-assisted alkaline hydrolysis. Furthermore, lactic acid and azelaic acid were successfully recovered. Therefore, the PLA-based copolyesters present high potential as substrates for engineering applications—such as printed electronics—owing to their rapid degradability and recyclability under microwave-assisted alkaline hydrolysis. These materials may serve as promising and competitive alternatives to conventional fossil-based, non-recyclable polymers commonly employed in such applications.

## Figures and Tables

**Table 1:** Alkaline hydrolysis of PLA, assisted with microwave irradiation, using different catalytic systems, at constant temperature and time.

Medium	Catalyst	Temperature (°C)	Time (min)	PLA Depolymerization (%)
NaOH 10% *w*/*v*	(2DME)-TPPB	80	5	56 ± 2
NaOH 10% *w*/*v*	HTMAC	80	5	64 ± 3
NaOH 10% *w*/*v*	HTMAB	80	5	69 ± 5
H_2_O	HTMAB	80	5	22 ± 1

**Table 2:** Effect of the alkaline concentration on the degradation of PLA, at constant temperature and time, using HTMAB as a phase transfer catalyst. The amount of the PLA samples was 0.5 g.

Medium	Catalyst (0.05 g)	Temperature (°C)	Time (min)	PLA Depolymerization (%)
NaOH 5% *w*/*v*	HTMAB	80	5	42 ± 3
NaOH 10% *w*/*v*	HTMAB	80	5	69 ± 5
NaOH 15% *w*/*v*	HTMAB	80	5	57 ± 3
NaOH 20% *w*/*v*	HTMAB	80	5	48 ± 2

**Table 3:** Effect of the amount of phase transfer catalyst on the degradation of PLA, at constant temperature and time, using HTMAB as a catalyst. The amount of the PLA samples was 0.5 g.

Medium	Catalyst (HTMAB)	Temperature (°C)	Time (min)	PLA Depolymerization (%)
NaOH 10% *w*/*v*	No catalyst	80	5	23 ± 4
NaOH 10% *w*/*v*	0.025 g	80	5	54 ± 6
NaOH 10% *w*/*v*	0.05 g	80	5	69 ± 5
NaOH 10% *w*/*v*	0.10 g	80	5	70 ± 2
NaOH 10% *w*/*v*	0.15 g	80	5	72 ± 3
NaOH 10% *w*/*v*	0.20 g	80	5	73 ± 2

**Table 4:** Effect of temperature on the depolymerization reaction of PLA. The amount of the PLA samples was 0.5 g.

Medium	Catalyst (0.05 g)	Temperature (°C)	Time (min)	PLA Depolymerization (%)
NaOH 10% *w*/*v*	HTMAB	60	10	61 ± 4
NaOH 10% *w*/*v*	HTMAB	80	10	86 ± 2
NaOH 10% *w*/*v*	HTMAB	100	10	93 ± 3
NaOH 10% *w*/*v*	HTMAB	120	10	97 ± 2

**Table 5:** Effect of time on the depolymerization reaction of PLA. The amount of the PLA samples was 0.5 g.

Medium	Catalyst (0.05 g)	Temperature (°C)	Time (min)	PLA Depolymerization (%)
NaOH 10% *w*/*v*	HTMAB	125	2	67 ± 3
NaOH 10% *w*/*v*	HTMAB	125	5	84 ± 2
NaOH 10% *w*/*v*	HTMAB	125	10	98 ± 2
NaOH 10% *w*/*v*	HTMAB	125	15	100

## Data Availability

NMR, DSC, enzymatic and depolymerization data are available on Zenodo (doi: https://zenodo.org/records/15423534, accessed on 1 May 2025).
